# Investigating the appropriate adenosine deaminase cutoff value for the diagnosis of tuberculous pleural effusion in a country with decreasing TB burden

**DOI:** 10.1038/s41598-022-11460-w

**Published:** 2022-05-09

**Authors:** Hyung Woo Kim, Kyung Hoon Kim, Ah Young Shin, Joon Young Choi, Joong Hyun Ahn, Ju Sang Kim, Woo Ho Ban, Jongyeol Oh, Jick Hwan Ha

**Affiliations:** 1 Division of Pulmonary and Critical Care Medicine, Department of Internal Medicine, Incheon St. Mary’s Hospital, College of Medicine, The Catholic University of Korea, Seoul, Republic of Korea; 2 Incheon Baek Hospital, Incheon, Republic of Korea; 3 Nasaret International Hospital, Incheon, Republic of Korea; 4 Incheon St. Mary’s Hospital, 56, Dongsu-ro, Bupyung, Incheon, 21431 Republic of Korea

**Keywords:** Tuberculosis, Respiratory tract diseases

## Abstract

As the burden of tuberculosis (TB) in South Korea decreases while that of malignancy increases with an aging society, the composition of etiology for pleural effusion is changing. The aim of this study was to investigate the diagnostic value of adenosine deaminase (ADA) for diagnosis of tuberculous pleural effusion (TPE) in this circumstance. Medical records of patients who underwent medical thoracoscopy from May 2015 to September 2020 in Incheon St. Mary Hospital, Korea were retrospectively reviewed. TPE was diagnosed if one of the following criteria was met: (1) granuloma in pleura, (2) positive TB polymerase chain reaction or culture in pleural fluid or tissue with non-specific pathologic findings in pleura, or (3) bacteriologically confirmed pulmonary TB with non-specific pathologic findings in pleura. A total of 292 patients, including 156 with malignant pleural effusion (MPE), 52 with TPE, and 84 with other benign effusion, were analyzed. Among 206 patients with lymphocyte dominant pleural effusion, the area under receiver characteristic curve of ADA for diagnosis of TPE was 0.971. The sensitivity and specificity of a current cutoff value of 40 IU/L were 1.00 and 0.61, respectively, whereas those of a raised cutoff value of 70 IU/L were 0.93 and 0.93, respectively. Among 54 patients with ADA levels of 40–70 IU/L, 30 (55.6%) patients were diagnosed as MPE, 21 (38.9%) as other benign effusion, and only 3 (5.6%) as TPE. Caution is needed in clinical diagnosis of TPE with current ADA cutoff value in countries with decreasing TB incidence, due to many false positive cases.

## Introduction

Tuberculosis (TB) is still a leading cause of death. In 2020, approximately 1.5 million deaths were attributable to TB^1^. Tuberculous pleural effusion (TPE) is the most common extrapulmonary tuberculosis (ETB) or the second most common ETB after TB lymphadenitis in many countries^2,3^. In South Korea, there were a total of 2,047 TPE patients in 2020, accounting for 10.3% of total notified TB patients and 43.4% of ETB patients^4^.

Although culture is the gold standard for the diagnosis of TB disease^5^, diagnosis of TPE cannot be made only with culture results as the sensitivity of pleural fluid culture is suboptimal – approximately 30% in previous reports^6^. Therefore, demonstration of granuloma in pleural tissue obtained with closed-needle biopsy had been the most widely used diagnostic method^7^. However, as it is an invasive procedure with potential risk of complications such as pneumothorax and hemothorax^8^, diagnostic approach measuring biomarker in pleural fluid that can reduce the need for such invasive procedures has been suggested^9^. The use of adenosine deaminase (ADA), a T-lymphocyte enzyme converting adenosine and deoxyadenosine to inosine and deoxyinosine, respectively, in the diagnosis of TPE was reported in 1978^10^. Since then, ADA has been a backbone of diagnostic flow^7^.

In high prevalence settings, patients with lymphocyte dominant exudative pleural effusion could be diagnosed as TPE when ADA of pleural fluid is over 40 IU/L based on excellent positive predictive value (PPV) of ADA in these settings^3^. However, in low prevalence settings, ADA can be used to exclude TPE due to its good negative predictive value (NPV)^11^. South Korea shows a decrease in TB incidence^12^. The TB incidence in 2000 was 96.3 per 100,000 population. It was decreased to 49.4 per 100,000 population in 2020^4^. Although the presumptive diagnosis of TPE with pleural ADA level over 40 IU/L and exclusion of other etiology was accepted in Korean guidelines for TB as in high prevalence settings^13^, the role of ADA should be re-established when its epidemiologic status changes.

With these backgrounds, the objective of this study was to investigate the diagnostic performance of pleural ADA level with various cutoff values. Unlike previous studies which included probable TPE patients who were diagnosed with elevated ADA level only^14–17^, we enrolled only patients who underwent medical thoracoscopy (MT) known to show the highest accuracy for diagnosis of TPE among various diagnostic tools with a role in the diagnosis of TPE being underscored^18–20^.

## Results

**Figure 1:**
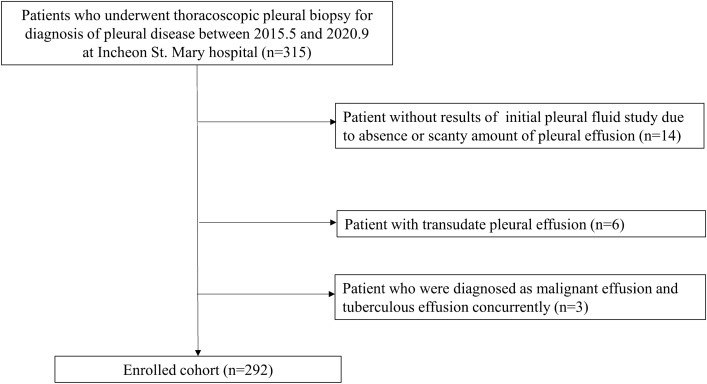
Patient enrolment flow chart.

A total of 292 patient who underwent MT were finally included (Fig. 1). Among them, 156 patients and 52 patients were diagnosed as MPE and TPE, respectively. Diagnosis of MPE was confirmed with tissue pathology of pleura in 151 (96.8%) patients (Table 1). The other five patients were diagnosed as MPE based on cytology of pleural fluid obtained before or during MT. The most common primary cancer site was lung (in 85.3% of total MPE patients). Among patients diagnosed as TPE (n = 52), 50 (96.2%) patients showed chronic granulomatous lesion in pleural pathology. Proportions of positive results for TB polymerase chain reaction (PCR) and acid-fast bacillus (AFB) culture were 46.2% (24/52) and 38.5% (20/52), respectively. In two patients, there was no pathologic evidence for TPE. However, TPE was confirmed by AFB culture positivity in pleural fluid (n = 1) or sputum (n = 1) without evidence for other pleural diseases (e.g., malignancy) in pleural fluid examination or MT. Baseline characteristics of enrolled patients by final diagnosis are described in Table 2. In patients with MPE, the proportion of females was higher than in patients with TPE or other benign effusion (42.9% vs. 25.0% or 28.6%, *p* = 0.018). Median age was lower in the TPE group than in the MPE or other benign effusion group (67 vs. 71.5 or 69.5 years, *p* = 0.094), although there was no statistically significant difference. There was no significant difference in comorbidities among MPE, TPE, and other benign effusion groups except for underlying malignancy, which was more prevalent in the MPE group than in the TPE or other benign effusion group (66.2% vs. 9.9% or 23.9%, *p* = 0.030).

**Table 1:** Diagnosis of malignant pleural effusion (n = 156), tuberculous pleural effusion (n = 52) and other benign effusion (n = 84).

1. Diagnosis of MPE (n = 156)			
(1) Methods of confirm diagnosis			
Tissue pathology (pleura)	Cell block (pleural fluid)^a^	N (%)	
Malignancy	Malignancy	101 (64.7)	
Malignancy	Atypical cells	17 (10.9)	
Malignancy	Other benign^b^	33 (21.2)	
Other benign	Malignancy	5 (3.2)	
**(2) Primary cancer site**			
Lung cancer	133 (85.3)		
Breast cancer	6 (3.8)		
Mesothelioma	4 (2.6)		
Primary peritoneal cancer	4 (2.6)		
Renal cell cancer	2 (1.3)		
Gastric cancer	2 (1.3)		
Pancreas cancer	2 (1.3)		
Cholangiocarcinoma	1 (0.6)		
Bladder cancer	1 (0.6)		
Endometrial cancer	1 (0.6)		

2. Diagnosis of TPE (n = 52)			
(1) Methods of confirm diagnosis			
Tissue pathology (pleura)	Tissue TB PCR (pleura)	AFB culture^a^ (pleural fluid or pleura)	N (%)
Chronic granulomatous inflammation	Positive	Growth of MTB	13 (25.0)
Chronic granulomatous inflammation	Positive	No growth	11 (21.2)
Chronic granulomatous inflammation	Negative	Growth of MTB	7 (13.5)
Chronic granulomatous inflammation	Negative	No growth	19 (36.5)
Other benign	Negative	Growth of MTB	1 (1.9)
Other benign	Negative	No growth	1^c^ (1.9)
**(2) Combined pulmonary TB**			
Bacteriologically confirmed pulmonary TB^d^	19 (36.5)		
Histologically confirmed pulmonary TB	1 (1.9)		
No pulmonary involvement	32 (61.5)		

3. Diagnosis of other benign effusion (n = 84)			
(1) Presumptive diagnosis			
Empyema/parapneumonic effusion	24 (28.6)		
Probable TB pleurisy^e^	3 (3.6)		
Asbestos-related plaque	1 (1.2)		
Chylothorax	1 (1.2)		
Chronic expanding hematoma	1 (1.2)		
IgG4-related pleuritis	2 (2.4)		
Lupus pleuritis	1 (1.2)		
Toxocariasis	1 (1.2)		
Idiopathic	50 (59.5)		

MPE, malignant pleural effusion; TPE, tuberculous pleural effusion; TB, tuberculosis; PCR, polymerase chain reaction; AFB, acid-fast bacillus; MTB, *mycobacterium tuberculosis.*

^a^Combined results from specimens obtained before thoracoscopy (by thoracentesis) and during thoracoscopy.

^b^Defined as all lesions without pathologic evidence of malignancy or tuberculosis.

^c^A case of pleural effusion combined with bacteriologically confirmed pulmonary tuberculosis whose cause for pleural effusion was not identifiable with pleural fluid exam or thoracoscopy.

^d^Cases with positive culture results or those with positive rapid diagnostic test such as Xpert MTB/RIF assay.

^e^Pleural effusion accompanying pulmonary tuberculosis, which was diagnosed clinically with radiologic findings, but not microbiologically confirmed.

**Table 2:** Baseline characteristics of enrolled patients.

	MPE (N = 156)	TPE (N = 52)	Other benign effusion (N = 84)	*p* value
**Sex**^a^				0.018
Male	89 (57.1)	39 (75.0)	60 (71.4)	
Female	67 (42.9)	13 (25.0)	24 (28.6)	
Age, median (IQR)	71.5 (63–79)	67 (55.5–76)	69.5 (59–79.5)	0.094
BMI, median (IQR)	23.4 (21.0–25.7)	22.4 (20.8–23.9)	23.1 (20.6–25.7)	0.080
**Smoking**^a^				0.550
Non-smoker	76 (48.7)	25 (48.1)	36 (42.9)	
Ex-smoker	62 (39.7)	17 (32.7)	34 (40.5)	
Current smoker	18 (11.5)	10 (19.2)	14 (16.7)	
**Comorbidities**				
Diabetes mellitus	34 (21.8)	8 (15.4)	23 (27.4)	0.258
Hypertension	67 (42.9)	18 (34.6)	37 (44.0)	0.506
Old TB	14 (9.0)	7 (13.5)	7 (8.3)	0.571
Coronary artery disease	9 (5.8)	4 (7.7)	4 (4.8)	0.750^b^
Heart failure	5 (3.2)	4 (7.7)	2 (2.4)	0.265^b^
Chronic kidney disease	5 (3.2)	5 (9.6)	6 (7.1)	0.154^b^
Liver cirrhosis	5 (3.2)	5 (9.6)	5 (6.0)	0.180^b^
Cerebrovascular accident	6 (3.8)	2 (3.8)	6 (7.1)	0.535^b^
Cancer	47 (30.1)	7 (13.5)	17 (20.2)	0.030

MPE, malignant pleural effusion; TPE, tuberculous pleural effusion; IQR, interquartile range; BMI, body mass index; TB, tuberculosis.

^a^Data are presented as numbers (columnar percent).

^b^Fisher’s exact test.

### Results of initial pleural effusion analysis

Results of initial pleural effusion analysis were compared between MPE, TPE, and other benign effusion group. As shown in Table 3, there was a significant difference in effusion/serum protein ratio (*p* < 0.001). It was lower in the other benign effusion group than in the MPE group (*p* = 0.002) or the TPE group in post-hoc analysis (median: 0.65 vs. 0.71 or 0.72, *p* < 0.001). In addition, serum-effusion albumin gradient was higher in the other benign effusion group than in the MPE group (*p* < 0.001) and the TPE group (*p* = 0.010) in post-hoc analysis (median: 1.1 g/dL vs. 0.8 g/dL or 0.8 g/dL, respectively). Differences in white blood cell (WBC) count and lymphocyte were statistically significant (*p* = 0.016 and *p* < 0.001, respectively). Patients with TPE showed higher WBC count and lymphocyte percentage than those with MPE (*p* = 0.006 and *p* < 0.001, respectively) in post-hoc analysis (median: 1400.5/μL vs. 787.5/μL, 80.5% vs. 61.6%, respectively). Pleural ADA level in the TPE group was higher than that in the MPE group (*p* < 0.001) or the other benign effusion group (*p* < 0.001) (median: 122 IU/L vs. 34 IU/L or 42 IU/L).

**Table 3:** Results of initial pleural effusion analysis.

	MPE (N = 156)	TPE (N = 52)	Other benign effusion (N = 84)	*p* value
Protein, effusion (g/dL)	4.8 (4.3–5.3)	5.1 (4.5–5.5)	4.7 (3.8–5.3)	0.018
Effusion/serum protein ratio	0.71 (0.65–0.75)	0.72 (0.67–0.78)	0.65 (0.59–0.74)	< 0.001
Albumin, effusion (g/dL)	2.8 (2.5–3.1)	2.7 (2.2–3.1)	2.4 (2.1–2.7)	< 0.001
Serum – effusion albumin gradient (g/dL)	0.8 (0.6–1.0)	0.8 (0.6–1.1)	1.1 (0.7–1.4)	0.001
LDH, effusion (U/L)	806 (465–1305)	613 (389.5–915)	561 (327–1069)	0.003
Effusion/serum LDH ratio	1.59 (1.09–2.70)	1.54 (1.02–2.43)	1.23 (0.68–2.56)	0.124
Glucose, effusion (mg/dL)	108 (82–132)	101 (84–117)	107 (93.5–137)	0.159
WBC count, effusion (/μL)	787.5 (335.5–1765)	1400.5 (558.5–3140.5)	886.5 (361–2144)	0.016
Neutrophil, effusion (%)	11.0 (4.1–23.3)	5.7 (2.0–23.0)	10.0 (3.5–33.7)	0.179
Lymphocyte, effusion (%)	61.6 (39.0–75.2)	80.5 (56.5–90.5)	67.7 (42.0–85.8)	< 0.001
Macrophage, effusion (%)	16.9 (10.0–29.6)	9.0 (4.0–15.0)	9.2 (5.1–18.5)	< 0.001
Eosinophil, effusion (%)	0.0 (0.0–2.7)	0.0 (0.0–0.0)	0.0 (0.0–1.1)	0.009
ADA, effusion (IU/L)	34 (26–47.5)	122 (91.5–156)	42 (26–65)	< 0.001

Data are median (interquartile range).

MPE, malignant pleural effusion; TPE, tuberculous pleural effusion; LDH, lactate dehydrogenase; WBC, white blood cell; ADA, adenosine deaminase.

### Diagnostic performance of ADA in lymphocyte dominant pleural effusion

**Figure 2:**
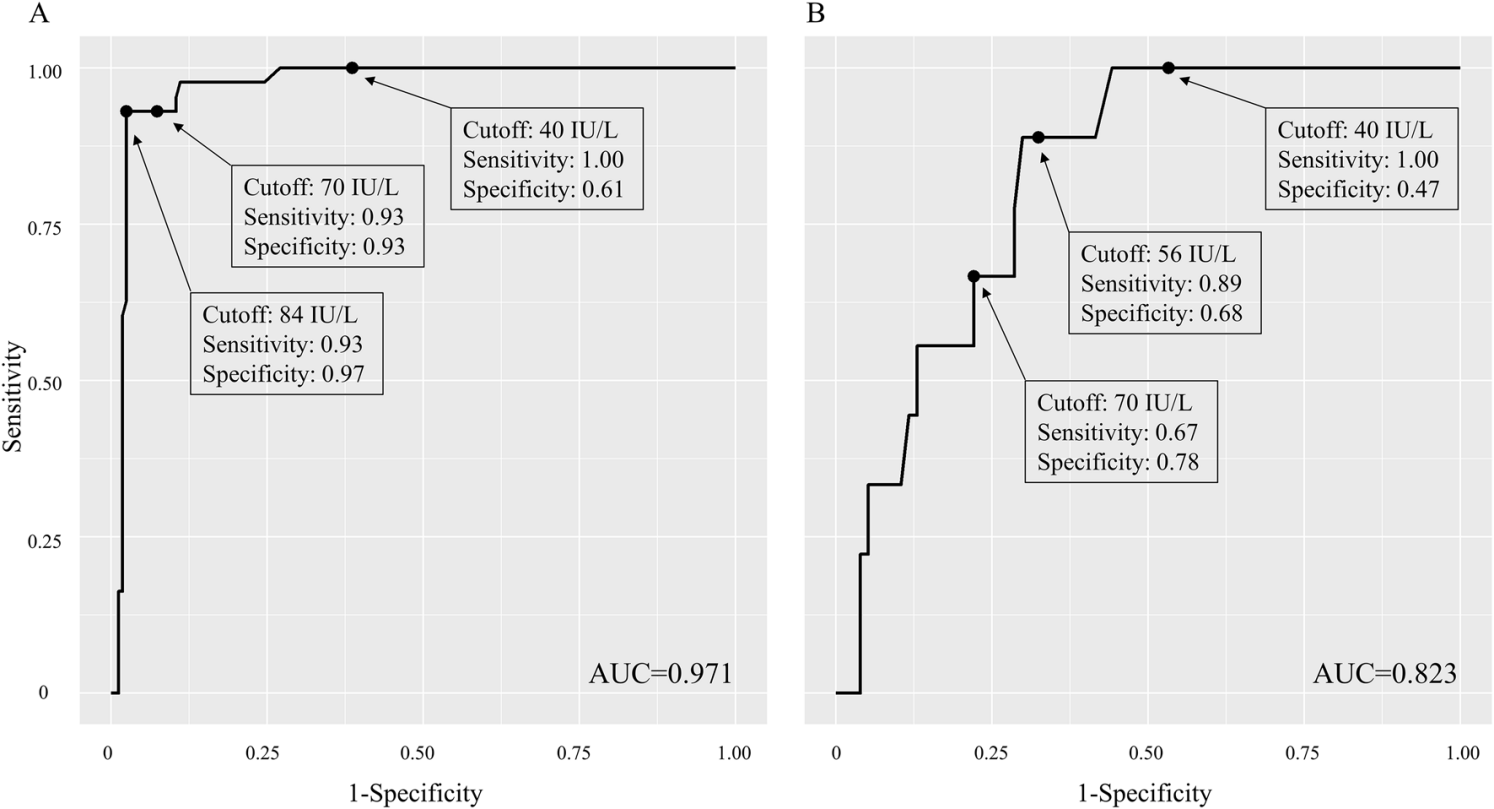
Receiver operating characteristic (ROC) curves presenting the performance of ADA in the diagnosis of TPE. ADA, adenosine deaminase; TPE, tuberculous pleural effusion; AUC, area under the curve Among patients with lymphocyte dominant exudative pleural effusion (panel **A**), ADA was an excellent tool for diagnosing tuberculous pleural effusion (AUC = 0.971). However, when applying the current cutoff value (40 IU/L), false positive cases presented with suboptimal specificity (0.61) could be a problem. Raising the cutoff value to 70 IU/L improved the specificity without sacrificing the sensitivity significantly. Among patients with non-lymphocyte dominant effusion (panel **B**), although ADA showed a lower diagnostic performance, it was a still good tool for diagnosing tuberculous pleural effusion (AUC = 0.823). With the current cutoff value (40 IU/L) and even the best cutoff value (56 IU/L) found in the ROC curve, specificity was still suboptimal when compared to that in lymphocyte dominant effusion.

**Figure 3:**
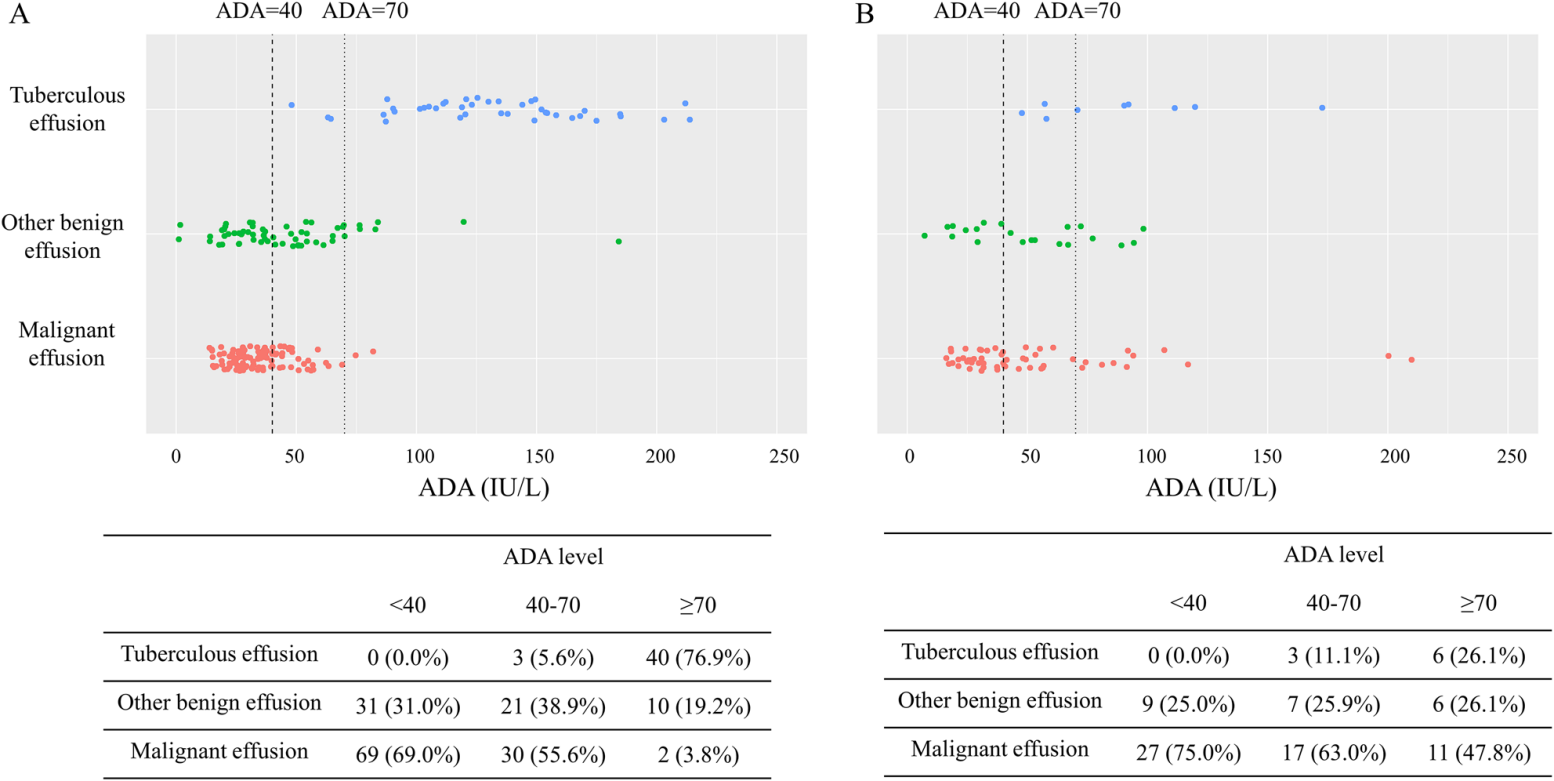
Distribution of ADA levels by the disease (tuberculous effusion, malignant effusion and other benign effusion). Lymphocyte dominant exudative pleural effusion was defined as a pleural effusion of which lymphocyte accounted for more than 50% of total leukocyte count. Patients with lymphocyte dominant exudative pleural effusion (panel **A**) were classified into three groups by ADA level with current cutoff value of 40 IU/L and raised cutoff value of 70 IU/L. There was no TPE case in the group presenting ADA level < 40 IU/L. In the gray zone (ADA level: 40–70 IU/L), the proportion of TPE was only 5.6%. Patients with non-lymphocyte dominant effusion (panel **B**) were also classified into three groups with cutoff values of ADA level 40 IU/L and 70 IU/L. There was no TPE case in the group presenting ADA level < 40 IU/L, as in patients with lymphocyte dominant pleural effusion. However, the proportions of TPE in the group presenting ADA level ≥ 40 IU/L and the group with ADA level ≥ 70 IU/L were relatively low when compared with patients with lymphocyte dominant effusion.

Among patients with lymphocyte dominant exudative pleural effusion (n = 206), ADA was an excellent tool for diagnosing tuberculous pleural effusion (area under the receiver operating characteristic (ROC) curve: 0.971) (Fig. 2A). The best cutoff value in our study was 84 IU/L. Diagnostic performances of pleural ADA level with current cutoff value (40 IU/L), raised cutoff value (70 IU/L), and the best cutoff value (84 IU/L) are presented in Table 4. With the current cutoff value, the sensitivity was excellent (1.00, 95% confidence interval (CI): 0.92–1.00). However, many false positive cases (n = 63) lead to suboptimal specificity (0.61 95% CI: 0.53–0.69). Raising the cutoff value up to 70 IU/L improved the specificity (0.93, 95% CI: 0.87–0.96) without sacrificing the sensitivity significantly (0.93, 95% CI: 0.81–0.99). Distributions of ADA levels in TPE, MPE, and other benign effusion groups are depicted in Fig. 3A. There was no TPE in patients presenting ADA level < 40 IU/L, reflecting excellent NPV of the current ADA cutoff value. However, in the gray zone (ADA level of 40–70 IU/L), the proportion of TPE was only 5.6%, which resulted in a low PPV of the current ADA cutoff value. In patients with ADA level ≥ 70 IU/L, 76.9% (40/52) had TPE.

**Table 4:** Diagnostic performances of ADA in lymphocyte dominant pleural effusion and non-lymphocyte dominant pleural effusion.

	TPE	Non-TPE	Sensitivity (95% CI)	Specificity (95% CI)	PPV (95% CI)	NPV (95% CI)
**Lymphocyte dominant pleural effusion**^a^						
**Current cutoff: 40 IU/L**						
ADA ≥ 40	43	63	1.00 (0.92–1.00)	0.61 (0.53–0.69)	0.41 (0.31–0.51)	1.00 (0.96–1.00)
ADA < 40	0	100				
**Raised cutoff: 70 IU/L**						
ADA ≥ 70	40	12	0.93 (0.81–0.99)	0.93 (0.87–0.96)	0.77 (0.63–0.87)	0.98 (0.94–1.00)
ADA < 70	3	151				
**Best cutoff: 84 IU/L**						
ADA ≥ 84	40	5	0.93 (0.81–0.99)	0.97 (0.93–0.99)	0.89 (0.76–0.96)	0.98 (0.95–1.00)
ADA < 84	3	158				
Non-lymphocyte dominant pleural effusion						
**Current cutoff: 40 IU/L**						
ADA ≥ 40	9	41	1.00 (0.66–1.00)	0.47 (0.35–0.58)	0.18 (0.09–0.31)	1.00 (0.90–1.00)
ADA < 40	0	36				
**Raised cutoff: 70 IU/L**						
ADA ≥ 70	6	17	0.67 (0.30–0.93)	0.78 (0.67–0.87)	0.26 (0.10–0.48)	0.95 (0.87–0.99)
ADA < 70	3	60				
**Best cutoff: 56 IU/L**						
ADA ≥ 56	8	25	0.89 (0.52–1.00)	0.68 (0.57–0.78)	0.24 (0.11–0.42)	0.98 (0.90–1.00)
ADA < 56	1	52				

ADA, adenosine deaminase; TPE, tuberculous pleural effusion; CI, confidence interval; PPV, positive predictive value; NPV, negative predictive value.

^a^Lymphocyte dominant pleural effusion was defined as a pleural effusion of which lymphocyte accounted for more than 50% of total leukocyte count in pleural effusion.

In a sensitivity analysis, excellent sensitivity (0.99, 95% CI: 0.95–1.00) with suboptimal specificity (0.66. 95% CI: 0.59–0.72) was identified with current ADA cutoff value (Table 5). Raising the cutoff value up to 70 IU/L improved the specificity (0.94, 95% CI: 0.90–0.97) without sacrificing the sensitivity significantly (0.92, 95% CI: 0.85–0.96), as in the original analysis.

**Table 5:** Diagnostic performances of ADA in lymphocyte dominant pleural effusion and non-lymphocyte dominant pleural effusion including additional TPE (n = 90) and MPE patients (n = 147) who did not undergo medical thoracoscopy and excluding the patients whose cause for pleural effusion remained unclear after medical thoracoscopy (n = 53).

	TPE	Non-TPE	Sensitivity (95% CI)	Specificity (95% CI)
**Lymphocyte dominant pleural effusion**^a^ **(n = 321)**				
**Current cutoff: 40 IU/L**				
ADA ≥ 40	111	71	0.99 (0.95–1.00)	0.66 (0.59–0.72)
ADA < 40	1	138		
**Raised cutoff: 70 IU/L**				
ADA ≥ 70	103	13	0.92 (0.85–0.96)	0.94 (0.90–0.97)
ADA < 70	9	196		
**Best cutoff: 76 IU/L**				
ADA ≥ 76	102	10	0.91 (0.84–0.96)	0.95 (0.91–0.98)
ADA < 76	10	199		
**Non-lymphocyte dominant pleural effusion (n = 155)**				
**Current cutoff: 40 IU/L**				
ADA ≥ 40	30	54	1.00 (0.88–1.00)	0.57 (0.48–0.66)
ADA < 40	0	71		
**Raised cutoff: 70 IU/L**				
ADA ≥ 70	27	25	0.90 (0.73–0.98)	0.80 (0.72–0.87)
ADA < 70	3	100		
**Best cutoff: 86 IU/L**				
ADA ≥ 56	25	16	0.83 (0.65–0.94)	0.87 (0.80–0.93)
ADA < 56	5	109		

ADA, adenosine deaminase; TPE, tuberculous pleural effusion; MPE, malignant pleural effusion; CI, confidence interval.

^a^Lymphocyte dominant pleural effusion was defined as a pleural effusion of which lymphocyte accounted for more than 50% of total leukocyte count in pleural effusion.

### Diagnostic performance of ADA in non-lymphocyte dominant pleural effusion

Among patients with non-lymphocyte dominant exudative pleural effusion (n = 86), the diagnostic value of ADA was lower than that in lymphocyte dominant pleural effusion (area under the ROC curve: 0.823) (Fig. 2B). The best cutoff value was 56 IU/L. Diagnostic performances of pleural ADA level with the current cutoff value of 40 IU/L, raised cutoff value of 70 IU/L, and the best cutoff value of 56 IU/L are presented in Table 4. With the current cutoff value, the sensitivity was excellent (1.00, 95% CI: 0.66–1.00). As in lymphocyte dominant pleural effusion, many false positive cases (n = 41) resulted in a suboptimal specificity (0.47, 95% CI: 0.35–0.58). Raising the cutoff value up to 70 IU/L improved the specificity to 0.78 (95% CI: 0.67–0.87) but impaired the sensitivity to 0.67 (95% CI: 0.30–0.93). Distributions of ADA levels in TPE, MPE, and other benign effusion groups are depicted in Fig. 3B. NPV of the current ADA cutoff level was excellent (1.00, 95% CI: 0.90–1.00) as in lymphocyte dominant effusion. However, PPV of both current ADA cutoff value and the raised cutoff value were suboptimal as proportions of TPE in the group presenting ADA level of 40–70 IU/L and the group with ADA level ≥ 70 IU/L were relatively low (11.1% (3/27) and 26.1% (6/23), respectively).

In a sensitivity analysis, the current cutoff value showed excellent sensitivity (1.00, 95% CI: 0.88–1.00) and suboptimal specificity (0.57, 95% CI: 0.48–0.66). Raising the cutoff up to 70 IU/L improved specificity (0.80, 95% CI: 0.72–0.87), but did not impaired sensitivity significantly (0.90, 95% CI: 0.73–0.98) owing to many pulmonary TB patients with neutrophil-dominant pleural effusion with ADA level over 70 IU/L who were additionally included in sensitivity analysis.

### Characteristics of high-ADA effusion

Comparison of clinical characteristics and thoracoscopic findings between MPE patients with low ADA level (< 40 IU/L) and those with high ADA level (≥ 40 IU/L) were presented in Table 6. Patients with high ADA level showed higher protein (p < 0.001) and LDH level (p = 0.001) in pleural fluid. All mesothelioma (n = 4) and gastric cancer (n = 2) patients showed high ADA level. Among the various thoracoscopic findings, high-ADA MPE was related with moderate to severe pleural adhesion (p < 0.001), hyperemia (p = 0.009), diffuse infiltration (p = 0.005) and pleural thickening (p < 0.001) suggestive of more severe pleuritis.

**Table 6:** Comparison of clinical characteristics and thoracoscopic findings between malignant pleural effusion patients with low ADA level (< 40 IU/L) and those with high ADA level (≥ 40 IU/L).

	MPE with low ADA (N = 96)	MPE with high ADA (N = 60)	*P* value
Age (years)	71 (63–79)	72 (64–79)	0.753
Sex: male	53 (55.2)	36 (60.0)	0.556
Sex: female	43 (44.8)	24 (40.0)	
Protein, effusion (g/dL)	4.5 (4.1–5.0)	5.1 (4.6–5.6)	< 0.001
Effusion/serum protein ratio	0.70 (0.64–0.73)	0.73 (0.68–0.79)	0.002
Albumin, effusion (g/dL)	2.7 (2.3–3.0)	3.1 (2.7–3.4)	< 0.001
Serum – effusion albumin gradient (g/dL)	0.9 (0.7–1.1)	0.7 (0.5–0.9)	0.003
LDH, effusion (U/L)	691.5 (442.8–1043.8)	1042.5 (546.8–1953.0)	0.001
Effusion/serum LDH ratio	1.44 (0.90–1.93)	2.41 (1.24–3.64)	< 0.001
WBC count, effusion (/μL)	750 (308.75–1750)	979.5 (395.5–1752.5)	0.169
Neutrophil, effusion (%)	11.0 (3.9–22.3)	11.0 (4.6–25.4)	0.370
Lymphocyte, effusion (%)	62.7 (47.7–75.1)	57.9 (29.1–75.3)	0.179
CEA, effusion (ng/mL)	96.0 (13.7–937.5)	134.6 (18.6–1004.8)	0.758
**Primary cancer site**			0.026
Lung cancer	81 (84.4)	52 (86.7)	
Breast cancer	4 (4.2)	2 (3.3)	
Mesothelioma	0 (0.0)	4 (6.7)	
Primary peritoneal cancer	4 (4.2)	0 (0.0)	
Renal cell cancer	2 (2.1)	0 (0.0)	
Gastric cancer	0 (0.0)	2 (3.3)	
Pancreas cancer	2 (2.1)	0 (0.0)	
Cholangiocarcinoma	1 (1.0)	0 (0.0)	
Bladder cancer	1 (1.0)	0 (0.0)	
Endometrial cancer	1 (1.0)	0 (0.0)	
**Thoracoscopic findings**			
Pleural adhesion			< 0.001
no or mild adhesion	83 (86.5)	34 (56.7)	
moderate to severe adhesion	13 (13.5)	26 (43.3)	
Pleural nodule	78 (81.3)	49 (81.7)	0.948
Hyperemia	28 (29.2)	30 (50.0)	0.009
Diffuse infiltration	28 (29.2)	31 (51.7)	0.005
Pleural plaque	36 (37.5)	24 (40.0)	0.755
Pleural thickening	16 (16.7)	39 (65.0)	< 0.001

Data are numbers (columnar percent) or median (interquartile range).

MPE, malignant pleural effusion; ADA, adenosine deaminase; LDH, lactate dehydrogenase; WBC, white blood cell; CEA, carcinoembryonic antigen.

Comparison between high and low ADA effusion among the other benign effusion group was presented in Table 7. As in MPE group, high-ADA was related with higher protein (p < 0.001) and LDH level (p = 0.003) in pleural fluid. Empyema or parapneumonic effusion accounted for 45.5% (20/44) of high-ADA other benign effusion, whereas 80% (32/40) of low-ADA effusion cases had no clear cause for pleural effusion even after MT. High-ADA other benign effusion was related with moderate to severe pleural adhesion (p = 0.004) and pleural thickening (p = 0.001).

**Table 7:** Comparison of clinical characteristics and thoracoscopic findings between other benign pleural effusion patients with low ADA level (< 40 IU/L) and those with high ADA level (≥ 40 IU/L).

	Other benign effusion with low ADA (N = 40)	Other benign effusion with high ADA (N = 44)	*p* value
Age (years)	69.5 (59.75–78.25)	69.5 (58.75–80)	0.922
Sex: male	25 (62.5)	35 (79.5)	0.084
Sex: female	15 (37.5)	9 (20.5)	
Protein, effusion (g/dL)	4.0 (3.3–4.8)	5.1 (4.6–5.5)	< 0.001
Effusion/serum protein ratio	0.63 (0.53–0.67)	0.71 (0.63–0.76)	< 0.001
Albumin, effusion (g/dL)	2.3 (1.9–2.6)	2.5 (2.2–2.8)	0.010
Serum–effusion albumin gradient (g/dL)	1.2 (0.9–1.5)	0.9 (0.7–1.3)	0.010
LDH, effusion (U/L)	415 (268.5–728.8)	796.5 (382.5–1593.3)	0.003
Effusion/serum LDH ratio	0.9 (0.6–1.5)	1.9 (0.9–3.4)	0.001
WBC count, effusion (/μL)	645.5 (303.5–1639.25)	1090.5 (400.5–2222.5)	0.144
Neutrophil, effusion (%)	9.0 (3.0–32.9)	10.0 (4.0–32.5)	0.566
Lymphocyte, effusion (%)	64.5 (52.5–89.2)	70.5 (37.8–82.0)	0.436
**Presumptive diagnosis**			< 0.001
Empyema/parapneumonic effusion	4 (10.0)	20 (45.5)	
Probable TB pleurisy^a^	0 (0.0)	3 (6.8)	
Asbestos-related plaque	1 (2.5)	0 (0.0)	
Chylothorax	1 (2.5)	0 (0.0)	
Chronic expanding hematoma	0 (0.0)	1 (2.3)	
IgG4-related pleuritis	1 (2.5)	1 (2.3)	
Lupus pleuritis	0 (0.0)	1 (2.3)	
Toxocariasis	1 (2.5)	0 (0.0)	
Idiopathic	32 (80.0)	18 (40.9)	
**Thoracoscopic findings**			
Pleural adhesion			0.004
no or mild adhesion	29 (72.5)	18 (40.9)	
moderate to severe adhesion	11 (27.5)	26 (59.1)	
Pleural nodules	5 (12.5)	4 (9.1)	0.730
Hyperemia	5 (12.5)	4 (9.1)	0.730
Diffuse infiltration	10 (25.0)	20 (45.5)	0.051
Pleural plaque	14 (35.0)	12 (27.3)	0.444
Pleural thickening	15 (37.5)	32 (72.7)	0.001

Data are numbers (columnar percent) or median (interquartile range).

ADA, adenosine deaminase; LDH, lactate dehydrogenase; WBC, white blood cell.

^a^Pleural effusion accompanying pulmonary tuberculosis, which was diagnosed clinically with radiologic findings, but not microbiologically confirmed.

**Figure 4:**
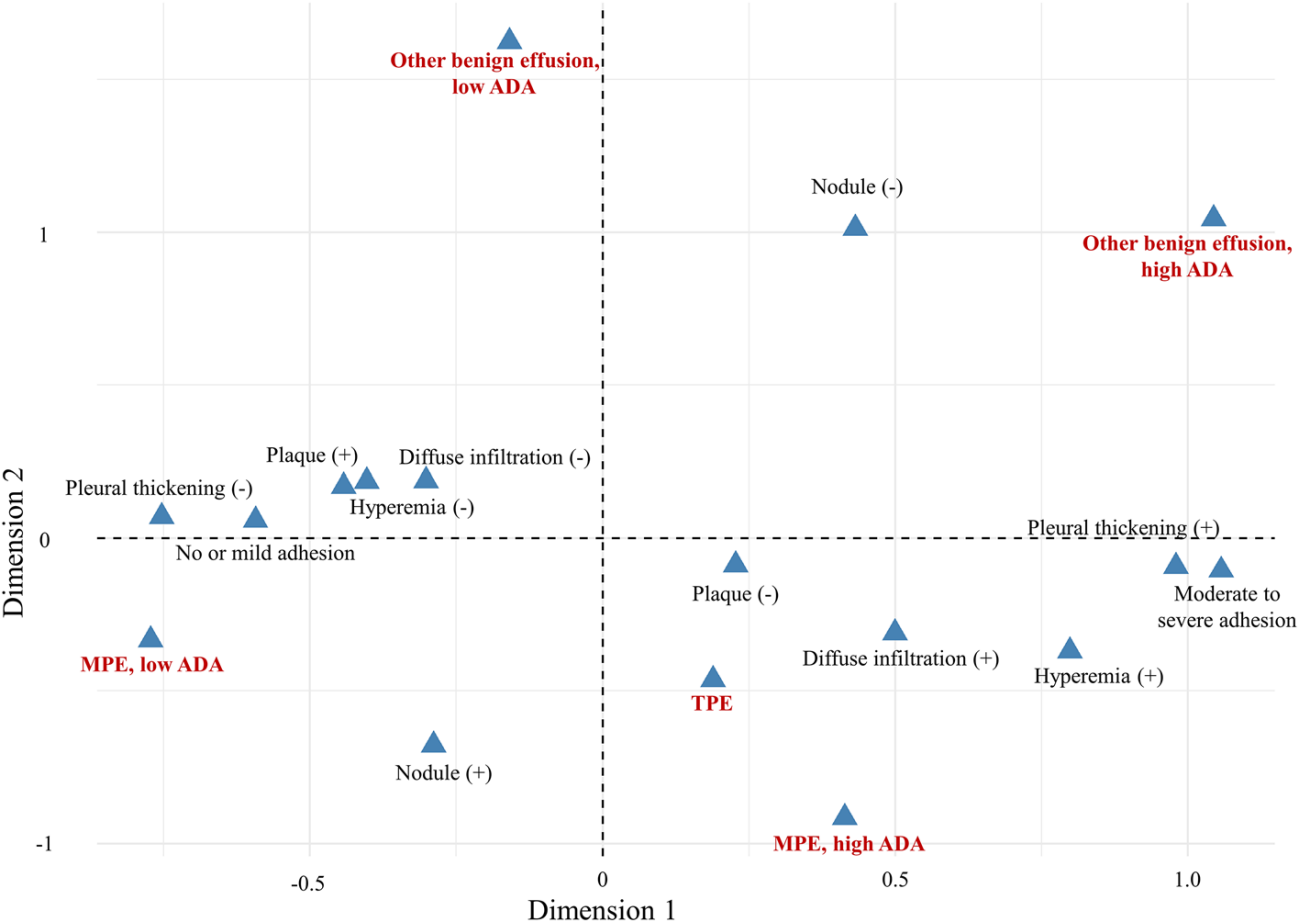
Plot of multiple correspondence analysis representing the relation between each feature of thoracoscopic findings and 5 subgroups by disease and ADA level. MPE, malignant pleural effusion; TPE, tuberculous pleural effusion; ADA, adenosine deaminase. Horizontal axis reflects the degree of pleural inflammation, right direction is related with more severe adhesion, pleural thickening, hyperemia and diffuse infiltration. In this plot, MPE patients with high ADA level and those with low ADA level showed disparate features in gross findings. Features of MPE patients with high ADA were similar with those of TPE patients. This finding implies that substantial proportion of MPE could be presented as severe pleural inflammation, which is related with elevated ADA level.

In a multiple correspondence analysis, MPE patients with high ADA level and those with low ADA level showed disparate features in gross findings (Fig. 4). Features of MPE patients with high ADA were similar with those of TPE patients, implying that substantial proportion of MPE could be presented as severe pleural inflammation, which is related with elevated ADA level.

## Discussion

In this study, we raised the problem of low PPV in diagnosis of TPE with current cutoff value of ADA. In our setting, among cases classified as gray zone with ADA level of 40–70 IU/L, only 5.6% cases were TPE. Therefore, caution is needed in clinical diagnosis of TPE with current ADA cutoff value in countries with decreasing TB incidence. However, we demonstrated the excellent NPV of ADA, which could be used as a good tool for the exclusion of TPE.

In Korean guidelines for TB, exclusion of other causes for pleural effusion is required before presumptive diagnosis of TPE with elevated ADA level only^13^. However, in real practice, methods for excluding other causes of pleural effusion are limited in many cases, especially among elderly patients who have various comorbidities raising the risk of general anesthesia required in thoracoscopic biopsy. MT, which does not require general anesthesia, is not yet widely performed in South Korea. Therefore, in many cases, physicians make a diagnosis of TPE only by pleural ADA level without thoracoscopic biopsy. This approach has a potential risk of delaying cancer diagnosis if the actual cause of pleural effusion is malignancy. In addition, in cases of other benign effusion, unnecessary anti-TB medication would be harmful to patients considering that the incidence of adverse effects during anti-TB treatment is relatively high in elderly patients^21^. In our study, patients who were diagnosed as other benign inflammatory effusion by thoracoscopic biopsy were followed up with chest X ray for a median of 246 days (range, 57 – 526 days). Although there were cases with relapsed pleural effusion, no patient was diagnosed as MPE or TPE afterwards. Ferrer et al. have reported 5-year follow-up results for 40 patients with idiopathic pleural effusion^22^. All patients enrolled in that study showed pleural ADA level below 43 IU/L. There was no incident TPE during the follow-up period. In our study, although the follow-up period was relatively short when compared with that of the previous study, our results suggested that even in patients with pleural ADA over 40 IU/L, the probability of TPE would be low if there was no evidence for TPE in thoracoscopy.

The diagnostic value of ADA is determined by TB prevalence in a local community^9,23^. In South Korea, the total of TPE cases was 1,409 in 2006. It was increased to 3,167 in 2011^4^. Thereafter, the number of TPE cases was decreased to 2,047 in 2020. However, the incidence of cancer as another major cause of pleural effusion is increasing continuously in elderly population in South Korea. The number of patients with lung cancer, a major cause for MPE, is continuously increasing from 9,815 cases in 2000 to 19,524 cases in 2018^24^. Considering current relative burden of TB and malignancy in elderly population, diagnosis of TPE with current ADA cutoff value can result in many false positive TPE cases. In many previous studies, ADA showed excellent sensitivity and specificity around the 90%^23,25–31^. In addition, it is well known that only 5% of MPE would show ADA level over 40 IU/L, especially among cases of lymphoma^7^. However, in our study, there were many false positive cases which lead to suboptimal specificity. Based on our findings, we carefully suggest that raising the cutoff value to reduce false positives is required, to preserve the diagnostic value of ADA in countries with decreasing TB burden and rapidly aging population. However, as this is the result of a single center study and discordant to the results of many previous studies demonstrating the excellent diagnostic value of ADA, further multicenter study is required to verify our suggestion.

More than one thirds of MPE patients showed ADA level over 40 IU/L in this study. We investigated the clinical characteristics of this high-ADA MPE patients and demonstrated that a substantial portion of MPE could be clinically presented as severe pleuritis. Based on findings in this study, we postulated that well-known risk factors for empyema or parapneumonic effusion such as poor dental hygiene, malnutrition, and alcohol abuse^32–34^ had contributed to the development of symptomatic pleuritis from the asymptomatic pleural metastasis and made patients visit a hospital, though we could not demonstrate that hypothesis due to the retrospective nature of this study. Such risk factors are related with socioeconomic status of the local community. Further studies on MPE involving several institutions in various local communities would be needed to investigate our hypothesis.

In this study, MT was performed to identify the cause of pleural effusion. This procedure has an advantage of avoiding general anesthesia while showing an excellent diagnostic performance as the tissue is sampled under direct visualization by the operator^35,36^. In a previous study, the sensitivity of MT was 100%^18^. However, thoracoscopy was recommended only in cases with undiagnosed or confusing etiology previously as it is an invasive procedure^7^. Decision models that can discriminate between MPE and TPE using pleural ADA and several other clinical variables have been investigated to reduce the needs for pleural biopsy^37,38^. However, in some cases of empyema, malignancy such as lymphoma and connective tissue disease can elevate ADA and result in false positive cases^2,7^. Thus, some experts have suggested that pleural biopsy should be considered when the patients’ clinical pictures is not typical for TPE with pleural effusion in the gray zone of pleural ADA level (40–70 IU/L)^2,7^. Our result further underscored the role of pleural biopsy among those in gray zone, as PPV of ADA in gray zone was suboptimal in our setting.

Previously, Lee et al. reported that pleural ADA could be lower than 40 IU/L in TPE patients who are in old ages or who are current smokers, which raise the number of false negative cases that can impair the sensitivity of ADA^14^. However, in our study, there was no false negative cases of TPE in the original analysis and only one false negative case was identified in the sensitivity analysis. Though current TB burden in South Korea is still higher than the countries with low TB burden, we demonstrated that the current cutoff value (40 IU/L) can be used for exclusion, not for definite diagnosis of TPE, based on excellent NPV, as in low prevalence settings^11^.

Diagnostic value of ADA in non-lymphocyte dominant effusion has not been clearly demonstrated yet. It has been suggested that TPE should not be ruled out in differential diagnosis for neutrophil dominant pleural effusion, especially when pleural ADA level is elevated^39^. We found that the probability of TPE could be clearly excluded in non-lymphocyte dominant effusion when the pleural ADA level was low (< 40 IU/L) based on the excellent NPV of ADA.

In our study, only patients who were histologically diagnosed were included, which could be a strength of this study. There were three cases diagnosed as MPE and TPE concurrently, which could underscore the importance of thoracoscopic biopsy. However, they were excluded from this study. Many previous studies on TPE have included patients with presumptive TPE diagnosis (lymphocyte dominant exudative effusion with high ADA levels (> 40 IU/L) in pleural fluid without any other cause or being resolved in response to anti-TB treatment)^14–17^. However, such cases were not included in our study as we presumed that by such definition, there could be false positive TPE cases (benign inflammatory lesion other than TB, actually) that could be resolved spontaneously without anti-TB treatment. In addition, for pulmonary TB cases with pleural effusion defined as TPE cases in previous studies as mentioned above, we did not include such patients unless they underwent thoracoscopic pleural biopsy. As a result, many pulmonary TB patients with ‘probable pleural’ involvement were not included in this study. This could be a shortcoming of our study as it could limit the generalizability of our findings to all TPE patients. To overcome this selection bias, we performed a sensitivity analysis including these TPE cases and MPE cases who did not undergo MT. The result was similar with that in the original analysis, which raised the robustness of our findings. However, as performing thoracoscopic pleural biopsy for all pulmonary TB patients with pleural effusion is not feasible or cost-effective, we cautiously selected the indication of thoracoscopy among such patients – those who were suspicious for other diagnosis, especially MPE. Although indication for thoracoscopy should be further investigated, we expect that our strategy is feasible in real clinical practice. In addition, Koh et al. have reported that TPE patients with pulmonary involvement shows higher ADA level than those without^40^. Based on this finding, we expect that the bias resulting from exclusion of many TPE cases with pulmonary involvement would be little.

As for MPE, we found out that there were more high-ADA MPE cases, which would become false positive cases for diagnosis of TPE, among the MPE patients who underwent MT than in those who did not undergo, though there was no statistical significance (60/156 (38.5%) vs 42/147 (28.6%), p = 0.069). There might be a chance that MT was performed with higher chances among such high-ADA MPE cases, which were presented like severe pleuritis, clinically. Another possible explanation would be that, when the patients who were suspicious of MPE showed high ADA level, MT would be more performed to rule out combined TB pleurisy. Despite this potential selection bias, the result of sensitivity analysis was similar with that in the original analysis. In addition, due to retrospective nature of this study, we could not investigate the patients’ symptom related with pleural effusion and risk factors for pleuritis, thoroughly. As mentioned above, characteristics of high-ADA MPE patients could be well investigated in a further prospective study.

In conclusion, caution is needed in clinical diagnosis of TPE with current ADA cutoff value in countries with decreasing TB incidence, due to many false positive cases. We carefully suggest that the current cutoff value of ADA for the diagnosis of TPE should be raised to reduce false positive TPE cases. However, the current cutoff value showed excellent NPV, demonstrating its usefulness in exclusion of TPE, as in low prevalence settings. In addition, the role of thoracoscopic biopsy should be underscored for cases with pleural ADA level in gray zone. Further studies are needed to verify our findings.

## Methods

### Patient selection

We retrospectively reviewed medical records of 319 patients who underwent MT for pleural biopsy at Incheon St. Mary Hospital, the Catholic University of Korea from May 2015 to September 2020. Exclusion criteria were as follows: (1) patients without results of initial pleural fluid study because of absence or scanty amount of pleural effusion; (2) patients with transudate pleural effusion; or (3) patients who were diagnosed as malignant pleural effusion (MPE) and TPE concurrently.

### Indication for pleural biopsy

Patients with exudative pleural effusion showing clinical presentation of empyema or parapneumonic effusion were referred to chest surgeon to determine the need for video-assisted thoracic surgery (VATS) decortication. They were not included in this study as both empyema and parapneumonic effusion were diagnosed clinically, not histologically. For the other patients with exudative pleural effusion, respiratory physicians determined whether or not to perform MT by following protocol. (1) When there are findings suggestive of malignancy on chest computed tomography (CT) scan, thoracoscopic pleural biopsy is performed when the operator expect that the risk of lung entrapment is low. (2) When there are findings suggestive of pulmonary tuberculosis on chest CT scan, then wait for the results of AFB stain and nucleic acid amplification test such as Xpert MTB/RIF (Cepheid, USA) with sputum or bronchial washing fluid which would come out within one or two days. If the results are negative, MT is indicated. If the results are positive, patients are diagnosed as tuberculous pleural effusion without thoracoscopy and initiate anti-TB treatment. However, among those pulmonary TB patients, MT is indicated when the probability of MPE could not be ruled out radiologically or the patients have clinical risk factors (e.g. underlying comorbidities of malignancy). (3) When there is no finding suggestive of malignancy or pulmonary TB, patients underwent abdomen and pelvis CT scan to find out malignancy or TB in abdomen and pelvis organs. If so, MT is indicated same as (1) and (2). (4) If there is no clue for malignancy and TB on chest, abdomen and pelvis CT scan, MT is indicated.

### Diagnosis of malignant effusion and tuberculous pleural effusion

The operator performed flex-rigid MT (LTF-240; Olympus, Tokyo, Japan). Pleural tissues were obtained with flexible biopsy forceps under direct visualization. To raise the diagnostic yield of pleural biopsy, the first five tissue samples were sent to a pathologist for frozen sectioning. If the pathologist confirmed malignancy or chronic granulomatous inflammation, at least 12 additional tissue samples were obtained. If the result was inconclusive or otherwise benign lesion, at least 20 additional tissue samples were taken. Tissue samples (at least one) were sent for tissue AFB culture without formalin fixation. Additionally, ‘targeted washing’, a procedure of washing around the target pleural lesion under direct visualization, was performed after tissue biopsy. Pleural fluids collected before and during MT were inoculated into BACTEC MGIT960 (Becton Dickinson, USA) and 3% OGAWA media (Eiken Chemical, Tokyo, Japan). AdvanSure TB/NTM real- time PCR (LG Lifescience, Seoul, Korea) was used for TB PCR. ADA level in pleural fluid was measured using automated colorimetric assay kit (Runpia Liquid ADA, Kyokuto Pharmaceutical Industrial Co. Ltd., Tokyo, Japan) according to manufacturer’s instruction. Thoracoscopic findings using a common standardized form were described by the operator just after the procedure and another respiratory physician reviewed this record. The adhesion grade was defined by an operator during MT. Mild adhesion was defined as the grade that complete adhesiolysis could be feasible during the procedure. Moderate adhesion was defined when exploration of some part of pleura was unfeasible due to pleural adhesion. Severe adhesion denoted the grade that exploration of most part of pleura was unfeasible, and when the operator concerned that diagnostic yield of MT might be impaired.

MPE was confirmed with malignant cells on histology samples (pleura) or cell blocks (pleural fluid collected before and during MT). A diagnosis of TPE was made for the following cases: (1) patients showing chronic granulomatous inflammation with or without caseous necrosis on histologic examination of pleura (for such cases, TB-PCR with formalin-fixed, paraffin-embedded pleura tissue was performed; regardless of tissue TB-PCR results, they were all defined as TPE unless there was an evidence for other granulomatous disease according to a previous report demonstrating that most pleural granulomatous inflammation was attributable to TB in South Korea^41^); (2) patients with positive results of either AFB culture or TB-PCR on pleural tissue or pleural fluid obtained before or during MT (for such cases, malignancy should be ruled out on histologic examination of pleura); (3) patients with bacteriologically confirmed pulmonary TB without evidence for other diseases on histologic examination of pleura. Other patients who did not meet the criteria of MPE or TPE were classified as otherwise benign effusion.

### Statistical analysis

Baseline demographic features and comorbidities were collected. Results of initial pleural fluid examination were compared among MPE, TPE, and otherwise benign effusion groups. For categorical variable, chi-square test or Fisher’s exact test was used. For continuous variable, Kruskal–Wallis test was used due to non-normality of variables. Additional post hoc analyses with Bonferroni method were performed. Patients were then classified by lymphocyte predominance, which was defined as the proportion of lymphocyte among total leukocyte count of pleural fluid being 50% or more. To demonstrate the value of ADA for the diagnosis of TPE, ROC curves were drawn for the lymphocyte dominant group and the non-lymphocyte dominant group. Best cutoff values of ADA in both groups were determined using Youden index. Diagnostic performance of ADA by various cutoff values, including the current cutoff value (40 IU/L) and the best cutoff value in our data, was presented with sensitivity, specificity, PPV, and NPV. Additionally, upper limit of gray zone (40–70 IU/L) suggested in previous studies^2,3,7^ was defined as the raised cutoff value (70 IU/L). The diagnostic performance by this cutoff point was also investigated.

To investigate any selection bias derived from including only the patients who underwent MT, those who did not underwent MT but diagnosed as TPE with microbiologic evidence for pulmonary TB and diagnosed as MPE with pleural fluid cytology during study period were additionally included and a sensitivity analysis was performed. Those whose cause for pleural effusion was unclear even after MT were excluded in the sensitivity analysis. Clinical characteristics and thoracoscopic findings between MPE patients with low ADA level (< 40 IU/L) and those with high ADA level (≥ 40 IU/L) were compared using chi-square test or Fisher’s exact test for categorical variables and Wilcoxon rank sum test for continuous variables. Also, those between other benign pleural effusion patients with low ADA level (< 40 IU/L) and those with high ADA level (≥ 40 IU/L) were compared. Relation between each feature of thoracoscopic findings and 5 subgroups (MPE, low-ADA MPE, high-ADA MPE, low-ADA other benign effusion, high-ADA other benign effusion) was visualized using multiple correspondence analysis.

All statistical analyses were performed using RStudio version 1.2.5033. Two-sided *P* < 0.05 was considered statistically significant.

### Ethics statement

Ethics approval was obtained from the Institutional Review Board (IRB) of Incheon St. Mary’s Hospital, Incheon, Korea (approval number: OC19RESI0047). Considering the retrospective design of this study, the requirement for informed consent was waived by the IRB. All research was performed in accordance with relevant guidelines and regulations.

## Data Availability

The datasets used and/or analysed during the current study are available from the corresponding author on reasonable request.
